# Gait Phase Estimation by Using LSTM in IMU-Based Gait Analysis—Proof of Concept

**DOI:** 10.3390/s21175749

**Published:** 2021-08-26

**Authors:** Mustafa Sarshar, Sasanka Polturi, Lutz Schega

**Affiliations:** 1 Health and Physical Activity, Department of Sport Science, Institute III, Otto von Guericke University Magdeburg, Zschokkestraße 32, 39104 Magdeburg, Germany; mustafa.sarshar@ovgu.de; 2 Medical Informatics, Institute of Medical Statistics, Computer and Data Sciences, Jena University Hospital, Bachstraße 18, 07743 Jena, Germany; sasanka.potluri@med.uni-jena.de

**Keywords:** inertial measurement unit, supervised deep learning

## Abstract

Gait phase detection in IMU-based gait analysis has some limitations due to walking style variations and physical impairments of individuals. Therefore, available algorithms may not work properly when the gait data is noisy, or the person rarely reaches a steady state of walking. The aim of this work was to employ Artificial Intelligence (AI), specifically a long short-term memory (LSTM) algorithm, to overcome these weaknesses. Three supervised LSTM-based models were designed to estimate the expected gait phases, including foot-off (FO), mid-swing (MidS) and foot-contact (FC). For collecting gait data two tri-axial inertial sensors were located above each ankle. The angular velocity magnitude, rotation matrix magnitude and free acceleration magnitude were captured for data labeling and turning detection and to strengthen the model, respectively. To do so, a train dataset based on a novel movement protocol was acquired. A validation dataset similar to a train dataset was generated as well. Five test datasets from already existing data were also created to independently evaluate the models. After testing the models on validation and test datasets, all three models demonstrated promising performance in estimating desired gait phases. The proposed approach proves the possibility of employing AI-based algorithms to predict labeled gait phases from a time series of gait data.

## 1. Introduction

By using modern technology, in particular the Inertial Measurement Unit (IMU), physical characteristics of human movement can be converted into a digital time series representation that allows us to extract meaningful information for gait analysis. In recent years, the clinical applications of IMUs, such as ambulatory monitoring, home-based rehabilitation, fall detection and the analysis of gait for disease detection have been growing in healthcare, aiming to enhance the quality of life and reduce the risk of injury, especially among patients [1,2].

In general, gait analysis of human locomotion is performed by capturing kinematic and kinetic parameters. To explore this periodic bodily movement (gait), it is necessary to detect gait phases. A normal gait cycle can be described by eight phases, which starts with the initial contact of one foot and ends with the terminal swing (the next initial contact) of the same foot, and these eight phases can be described by two main periods, (1) stance period, and (2) swing period [3]. One constraint of understanding this gait cycle would be the fact that only the first initial contact, also known as heel strike (HS), would be considered as the start of each gait cycle, and the events before the first HS stay unknown. However, walking may start first by elevating one foot, known as toe off (TO) followed by the forward movement and replacement of the same foot, known as the swing phase, where mid-swing (MidS) takes place, and later ends by HS. Furthermore, according to [4], the analysis of gait initiation, which in most studies has been overlooked, may provide health care practitioners with essential information to identify cognitive and motor impairments at very early stages of the disorder related to mobility impairments.

Although the purpose of quantification of human movement by converting the continuous biomechanical signal to discrete data is straightforward, the methods and algorithms for extracting accurate and useful data still requires complicated calculations [5]. To the best of the author’s knowledge, there are multiple methods for IMU-based gait analysis to estimate the gait phases and steps. Most common approaches are based on filtering, thresholding and peak detection algorithms, automatically or semi-automatically, in order to find the desired gait phases [6]. There are also other techniques trying to match step templates. These templates are set manually or gained from gait data of an individual or from a collection of training data [6]. Furthermore, Artificial Intelligence (AI) or adaptive algorithms have also been employed in IMU-based gait analysis in recent years. Reference [7] has already reviewed AI-based algorithms, such as the Hidden Markov model (HMM), Bayesian model, and support vector machine (SVM). Accordingly, AI can improve the gait analysis by adapting its decision-making technique based on the variability of gait patterns. Other AI techniques have been applied to different measurements. For example, artificial neural networks (ANN) for stride length estimation in [8], machine learning classifiers, such as k-Nearest Neighbor (k-NN), SVM, Naive Bayes (NB), and decision trees (DTs) to study the gait behavior of Parkinson’s patients in [9], a Recurrent Convolutional Neural Network (RCNN) model for gait-based user authentication introduced by [10], Long-Short Term Memory (LSTM) algorithm for normal and pathological gait classification presented by [11] and SVM for gait classification of young and elderly people proposed by [12].

Although AI-based algorithms, in particular LSTM, are strongly capable of exploring meaningful information from a long-term series of data, feature engineering applied for gait phase detection is challenging and requires field-specific expertise.

The aim of this study is (1) to propose a novel approach to create a train dataset for gait phase detection, (2) to demonstrate a simple and efficient approach based on an LSTM model to estimate gait phases from all steps, including gait initiation while walking straight forward, and (3) to assess the scalability of the model by evaluating its performance on multiple unseen validation and test datasets.

## 2. Materials and Methods

### 2.1. Data Analysis and Setup

This work is conducted as a part of an ongoing project, Gait control—visuo-motor interactions and plasticity in glaucoma (reg. no.: DRKS00022519). All data analysis and signal processing algorithms were performed in Python programming language (Python Software Foundation. Python Language Reference, version 3, https://www.python.org (accessed on 1 March 2021)). Moreover, for processing the signals and training the model, “scipy” (https://scipy.org/ (accessed on 1 March 2021)), “pandas” (https://pandas.pydata.org/ (accessed on 1 March 2021)) and “TensorFlow 2” (https://www.tensorflow.org/ (accessed on 1 March 2021)) via Keras APIs (“https://keras.io/ (accessed on 1 March 2021)”) were employed. Source code is available in GitHub repository (https://github.com/mustafa-sarshar/SmartGait (accessed on 11 August 2021)), please refer to SmartGait_01 directory to get the codes and run and test the models presented in this work.

**Figure 1:**
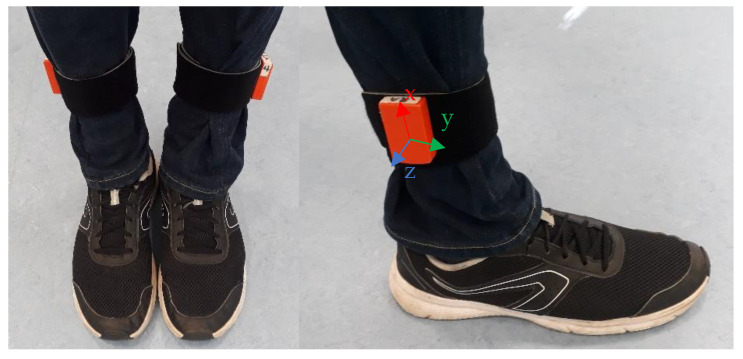
The placement of the sensors on each leg.

Two tri-axial inertial sensors (XSENS MTw Awinda, Xsens Technologies B.V., Enschede, The Netherlands) were employed for gait analysis. The XSENS MTw Awinda is a miniature wireless IMU, which provides the users with highly accurate real-time 3D orientation signals. This state-of-the-art motion tracker has a 3D gyroscope, a 3D accelerometer as well as a 3D magnetometer. The output signal is already optimized by XSENS Kalman Filter to guarantee high performance measurement with no data loss while capturing human-relevant motions [13]. For this study, the sensors were placed above the right and left ankles (Figure 1). The sampling rate was set to 100 Hz.

Primary kinematic parameters, including 3D angular velocity signals (Gyr_X, Gyr_Y, and Gyr_Z) for labeling the gait phases, as well as rotation matrix “Mat [1][1]” (RotMat), also known as Direction Cosine Matrix to detect turning and 3D free acceleration signals (FreeAcc_X, FreeAcc_Y, FreeAcc_Z) to strengthen the gait phase estimation, were gained from XSENS MT-Manger software v4.6 (https://www.xsens.com/software-downloads (accessed on 1 March 2021)).

### 2.2. Data Collection

#### 2.2.1. Train Dataset

To acquire the train dataset, a list of multiple tasks (movement protocol) was prepared in a way that the model will learn multiple different over-ground walking styles and will become compatible with different lower-body movements at different walking speeds. To do that, two of our research group members voluntarily performed the related tasks while wearing the sensors. These tasks were as follows:

- Task 1: walk straight forward at a self-selected convenient pace for about 10 m (without running or jumping) and then stop, turn and return at the same speed. The task would be repeated for two more round-trips, one at a faster and one at a slower speed compared to the first round-trip. This would be considered as one trial. Each subject performed two trials.
- Task 2: turn 360 degrees while taking small steps without changing the place. This task was performed at a self-selected convenient pace and then repeated for two more tries, one at a faster and one at a slower speed compared to the first try. This would be considered as one trial. Each subject completed two trials.
- Task 3: the first task was repeated; however, the subject was asked to change the speed while walking. Each subject performed one trial.
- Task 4: stepping with both legs. This task was performed first at a self-selected convenient pace (two steps with each leg) without moving or changing the place. After waiting for two seconds the task would be repeated for two more tries, one at a faster and one at a slower pace compared to the first try. This would be considered as one trial. Each subject completed one trial.
- Task 5: the 4th task was repeated; however, the subject was asked to slightly step forwards and backwards while performing the task without changing the place and turning. Each subject performed one trial.
- Task 6: walk forward while turning. The subject was asked to stand at one side of the walking track just before the end of the track and then start turning 180 degrees while walking to the other side and then stop. After two seconds they returned at the same speed. After that, the task was repeated for two more round-trips, one at a faster and one at a slower pace. This would be considered as one trial. Only one subject completed this task for two trials.

#### 2.2.2. Validation Dataset

In order to generate the validation dataset, the abovementioned tasks were repeated by two other colleagues, voluntarily. However, the tasks were performed for a shorter duration and there were less trials compared to the train dataset, as follows:

- Task 1 and 2: each subject completed the same task for only one trial.
- Task 3: each subject performed this task for only one trial; however, the subjects were asked to complete only one round-trip.
- Task 4, 5 and 6: each subject completed the same task for only one trial.

#### 2.2.3. Test Dataset

For test datasets, data were collected from (1) the abovementioned project and (2) from another ongoing project, “A multimodal intervention approach for cross-sectoral care of degenerative diseases of the spine, MultiMove” (reg. no.: DRKS00021696). Therefore, the test datasets included gait data from healthy individuals, glaucoma patients and chronic low back pain (cLBP) patients, who were asked to walk over-ground. In total, five test datasets were generated as described below:

1. Test_1_H and Test_1_G, including gait data from healthy individuals and glaucoma patients, respectively. Subjects walked forward at their self-selected convenient pace for about 13 m and then turned while walking to the left and walked back to the start position.
2. Test_2_H, Test_2_G, including gait data from healthy individuals and glaucoma patients, respectively. Subjects walked first about 10 m straight forward at their self-selected convenient pace, then stopped and turned 180 degrees and returned at their self-selected slow pace, then stopped and turned 180 degrees and walked again 10 m forward at their self-selected fast pace and finally stopped and turned 180 degrees.
3. Test_3_cLBP, including gait data from cLBP patients. Subjects walked straight forward over-ground at their self-selected convenient pace. The walking track was 15 m long and subjects were asked to walk about 30 s without stopping and continue walking while turning at each end of the track.

As a result, the models in each epoch would be trained and tested by using datasets having gait data from similar activities and then later the scalability of them would be assessed by using real unseen gait data from subjects walked over-ground.

### 2.3. Data Processing

#### 2.3.1. Data Cleaning and Signal Filtering

The data was processed first by extracting the required data from the datasets and then dropping unnecessary columns. Next, data imputation was performed by interpolating the data using the “pad” method from “pandas” library; however, no missing values were reported. Later, the outliers were detected and removed by considering the valid range for accelerometer and gyroscope signals, ±160 m/S^2^ and ±2000 degree/s, respectively, according to the guidelines of the XSENS company for MTw sensors mentioned by [13]. Later, the signals were smoothed by using the Gaussian smoothing filter method (full-width at half-maximum = 9) from the “scipy” library.

#### 2.3.2. Feature Extraction

In several studies, the angular velocity signal was employed to detect the gait phases [14,15,16] due to the fact that the signal gained from the gyroscope is reliable for gait phase detection among healthy as well as unhealthy people. According to the findings of [15], the angular velocity parallel to the mediolateral axis (perpendicular to the sagittal plane) can be used to detect the peaks during TO and HS. Moreover, based on the orientation and position of our sensors, this signal would be equal to the signal gained from Gyr_Z from our sensors. However, this signal may look different if we change the orientation of the sensors. Therefore, to gain invariant signals independent of the sensor’s orientation, kinematic signals including angular velocity magnitude (*AngVelMag*) and free acceleration magnitude (*FreeAccMag*) were calculated by using Equations (1) and (2), respectively. The rotation matrix magnitude (*RotMatMag*) was also calculated by using Equation (3).

(1) $$AngVelMag=\sqrt{Gyr\_X^{2}+Gyr\_Y^{2}+Gyr\_Z^{2}}$$

(2) $$FreeAccMag=\sqrt{FreeAcc\_X^{2}+FreeAcc\_Y^{2}\;FreeAcc\_Z^{2}}$$

(3) $$RotMatMag=\sqrt{left\;leg\;RotMat^{2}+right\;leg\;RotMat^{2}}$$

#### 2.3.3. Feature Labeling

**Figure 2:**
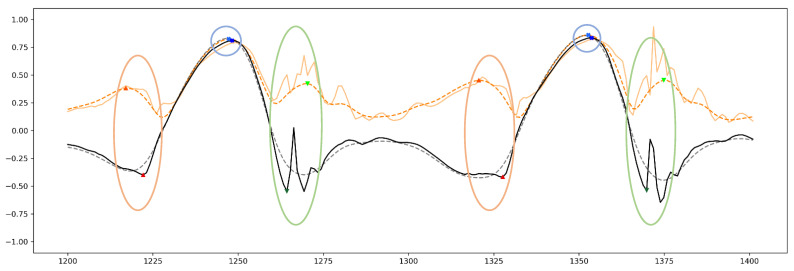
An example of the angular velocity signal used for labeling the gait phases. These signals are scaled from −1 to 1. The black solid line represents Gyr_Z, the gray dashed line represents smoothed Gyr_Z, the solid orange line represents AngVelMag, and the dashed orange line represents smoothed AngVelMag. HS, MidS and TO are shown in dark red, dark blue and dark green markers on Gyr_Z based on the concept of [15] and FO, MidS and FC are highlighted with light red, light blue and light green markers on smoothed AngVelMag according to our approach for labeling gait phases.

The exact time points of TO and HS based on Gyr_Z have already been explored and validated by footswitches (as a gold standard) located under toe and heel of each foot in [15]. In our study, a very similar concept was used for labeling the data and defining gait phases. However, we used smoothed AngVelMag instead of Gyr_Z, and labeled the data for FO and FC instead of TO and HS as seen in Figure 2. To do so, FO was considered as the time point when the foot leaves the ground. Therefore, the FO may appear some frames prior to the TO defined by [15], since the smoothed AngVelMag can be influenced by all signal peaks when the foot is taken off the floor. Additionally, the FC was defined as the time point that the foot contacts the floor again. Therefore, FC may appear some frames later than the HS presented by [15], because of the same reason mentioned for FO when the foot hits the floor. Moreover, the data was labeled for MidS as well. For this purpose, we considered the local maxima of the smoothed AngVelMag between FO and FC as the time point for MidS. To encode the data, gait phases were labeled as 1 and non-gait phases as 0. To do that, one separate labeled feature for each gait phase comprising only 0 or 1 values were added to the datasets.

**Figure 3:**
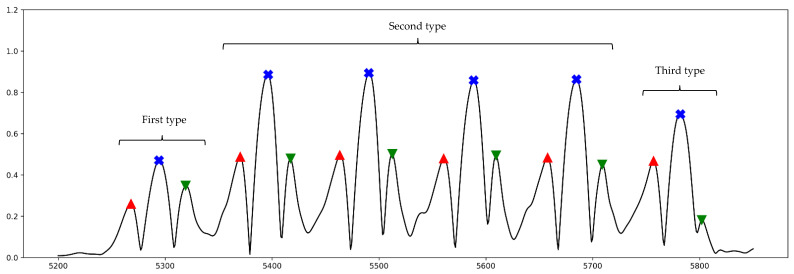
This figure shows three different step types, first step of walking (**first type**), four steps while in steady state (**second type**), and the final step of walking (**third type**). Red markers: FO; blue markers: MidS; green marker: FC. The black line represents the smoothed AngVelMag signal.

As seen in Figure 3, there were in general three different types of steps in our gait data with regard to the behavior of signal and its amplitude at FO, MidS, and FC. First type is the first step of walking, which takes place before any other step where in some cases the amplitude of FO is lower than others. The second type includes the steps after the first step until one step prior to stopping. In general, these steps are seen when the person reaches the steady state of walking. The signal amplitude of FO in most cases is higher than others. During the steady state of walking, all three gait phases maintain similar amplitude till the person stops. Finally, the last step occurs each time the person stops or terminates walking for longer than a couple of seconds, where FO still has the highest amplitude while FC has a very low amplitude compared to others. In some cases, it can be challenging to detect FC from the final step.

**Figure 4:**
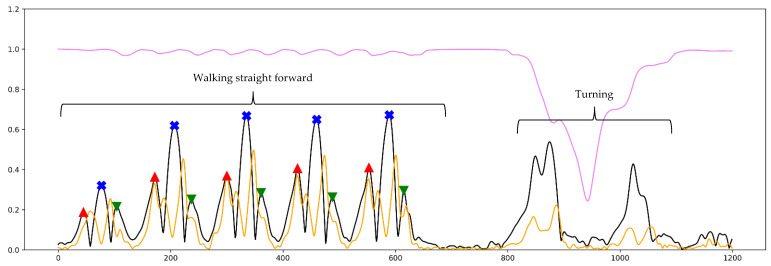
This figure illustrates a short part of the train dataset. Black line: smoothed AngVelMag; orange line: smoothed FreeAccMag; violet line: smoothed RotMatMag; red markers: labeled FO; blue markers: labeled MidS; green marker: labeled FC.

Moreover, steps were divided into valid and invalid steps, and only valid steps were included in gait phase detection. A valid step was defined as a step while the person is walking straight forward without having excessive jerky moves (the steps in task 1 and 3 of the movement protocol). However, an invalid step was defined as a step while the person is not walking straight forward or turning to the right or left, or stepping without moving or without changing the place (the steps in task 2, 4, 5 and 6 of the movement protocol). These steps were not considered to be valid steps; therefore, they were not labeled for FO, MidS, and FC. Figure 4 outlines a short period of train dataset where only valid steps are labeled for gait phase estimation and invalid steps are ignored.

### 2.4. Neural Network Architecture

#### 2.4.1. Background

The recurrent neural network (RNN) is capable of working with time series. To the best of our knowledge, LSTM is a type of RNN and is developed to solve long-time-lag problems of gradient-based methods. Moreover, it is designed specifically to analyze long time series and to overcome the error backflow problems, known as blowing up and vanishing. It is capable of learning the characteristics of complex data faster than other RNN models [17]. Further, this technique has been employed for gait analysis and has become popular among scientists in recent studies [18,19,20].

#### 2.4.2. Design

**Figure 5:**
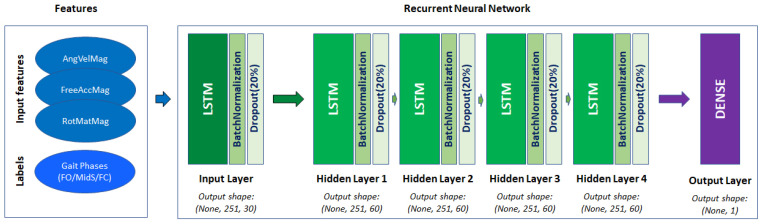
The structure of the Recurrent Neural Network.

As mentioned before, the models were designed based on the LSTM algorithm using Keras API from the TensorFlow 2 package in Python. For each gait phase, one LSTM-based regression model was designed, LSTM_FO, LSTM_MidS and LSTM_FC to detect FO, MidS and FC, respectively. The data from train and validation datasets were vectorized first by being reshaped to a tensor of size (None, 251, 3), having a fixed length sequence of 251 frames and 3 input features in a way that each sequence of input features “X” should estimate whether the desired gait phase takes place at the middle of the sequence (125th frame) or not. This would then generate the labeled feature “y”, having values of 0 or 1. After testing different hyperparameters, we decided to design each model as follows: one input LSTM layer (units = 30), followed by three hidden LSTM layers (units = 60,), one more hidden LSTM layer (unit = 30) and finally one output Dense layer (units = 1, activation = sigmoid). Each input and hidden layer was followed by one BatchNormalization layer (momentum = 0.99, epsilon = 0.001) and one Dropout layer (rate = 0.2). Each model was compiled by an Adam optimizer (learning rate = 0.0001) and binary cross-entropy loss function. Afterwards, each model was trained for 100 epochs (shown in Figure 5).

## 3. Results

### 3.1. Datasets

Table 1 outlines an overview of all datasets and highlights some characteristics of the subjects. As seen in Table 1, Test_3_cLBP was the largest dataset and Test_1_G was the smallest one. Subjects in train and validation datasets were younger and lighter than subjects in test datasets. Each train and validation dataset comprised gait data from only two subjects while test datasets comprised gait data from 16 healthy individuals, 11 glaucoma patients and 37 cLBP patients.

### 3.2. The Performance of Each Model

Each model was trained by using the Mirrored Strategy method provided by TensorFlow; therefore, all available CPU cores and GPUs would be employed for the training process. We used a Notebook with Intel Core i9 10th GEN CPU and a CUDA-enabled GPU (GeForce RTX™ 2080). Each epoch took on average 940 s. As a result, it took approximately 26.1 h to train each model.

Table 2 shows the accuracy and loss of each model during the training process. All three models achieved the same level of accuracy on the train dataset, 0.9981; however, the loss was slightly different among the three models, where the best loss was achieved by LSTM_FO, 0.0071, followed by LSTM_FC, 0.0072, and the weakest gained by LSTM_MidS, 0.0080. The models also gained the same accuracy on the validation dataset, 0.9977, while having different losses, where the lowest was achieved by both LSTM_FO and LSTM_FC, 0.0098, and the highest gained by LSTM_MidS, 0.0102.

Table 3 illustrates the performance of each model by considering their performances on unseen test datasets. On average, all models achieved the same accuracy, 0.9945, while having different losses, where the lowest loss was achieved by LSTM_FO, 0.0249, followed by LSTM_MidS, 0.0261, and the highest gained by LSTM_FC, 0.0290. Moreover, the highest accuracy achieved equally by all three models was 0.9960 on Test_2_G, and the lowest accuracy gained equally by all three models was 0.9935 on Test_3_cLBP. The best loss achieved by LSTM_FO was 0.0186 on Test_2_G, and the weakest loss gained by LSTM_FC was 0.0395 on Test_1_G. Therefore, the performance of models was clearly better on the validation dataset compared to test datasets.

**Figure 6:**
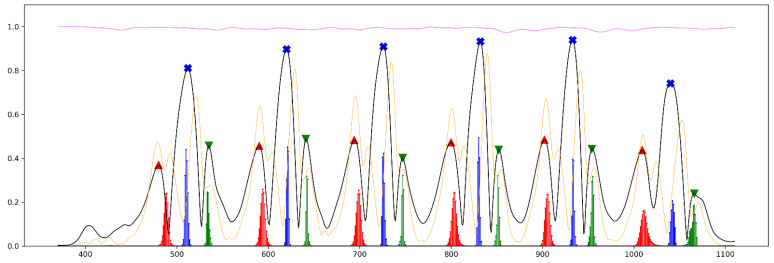
A short part of the prediction results from Test_2_H. This figure demonstrates six steps from the beginning of walking till the last step before turning at the end of the walking track. Black line: smoothed AngVelMag; orange line: smoothed FreeAccMag; violet line: smoothed RotMatMag, red markers: labeled FO; red vertical lines: predictions for FO; blue markers: labeled MidS; blue vertical lines: predictions for MidS; green marker: labeled FC; green vertical lines: predictions for FC.

**Figure 7:**
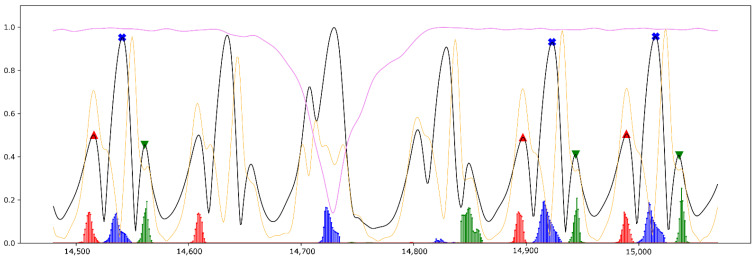
A short part of the evaluation results from Test_1_G. This figure shows six steps while walking straight forward, turning at the end of the walking track, and then walking back. Black line: smoothed AngVelMag; orange line: smoothed FreeAccMag; violet line: smoothed RotMatMag, red markers: labeled FO; red vertical lines: predictions for FO; blue markers: labeled MidS; blue vertical lines: predictions for MidS; green marker: labeled FC; green vertical lines: predictions for FC.

**Figure 8:**
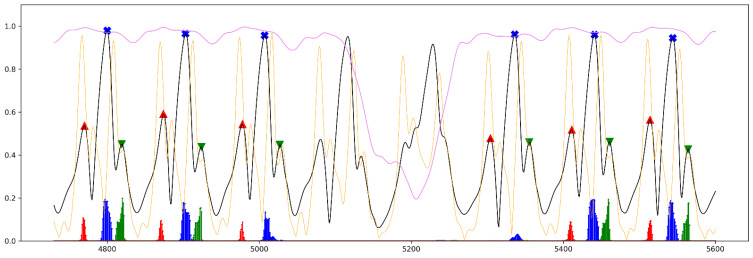
A short part of the evaluation results from Test_3_cLBP. This figure depicts eight steps while walking straight forward, turning at the end of the walking track, and then walking back. Black line: smoothed AngVelMag; orange line: smoothed FreeAccMag; violet line: smoothed RotMatMag, red markers: labeled FO; red vertical lines: predictions for FO; blue markers: labeled MidS; blue vertical lines: predictions for MidS; green marker: labeled FC; green vertical lines: predictions for FC.

Figure 6, Figure 7 and Figure 8 illustrate the prediction results gained from all three regression models from the short part of Test_2_H, Test_1_G and Test_3_cLBP, respectively. In all figures, the results of the predictions are plotted together as well as all input and labeled features in different colors scaled from 0 to 1 for comparison purposes.

Figure 6 illustrates a short part of gait data from Test_2_H from the first step till the last step before turning to the left. Accordingly, the person reaches a steady state of walking after the second step. At the first two steps, the amplitude of smoothed AngVelMag at all FOs is lower than its amplitude at all FCs. This phenomenon changes from third step till fifth step, where its amplitude at all FOs is lower than its amplitude at all FCs. At the last step, its amplitude at FO does not dramatically change, however it drops sharply at FC. Moreover, its amplitude at MidS was to a great extent more consistent and higher compared to its amplitude at other gait phases. Further, LSTM_FO and LSTM_FC predicted all FOs and FCs correctly, while LSTM_MidS had one incorrect prediction. At the first step, LSTM_MidS predicted the MidS correctly, however the probability of its best prediction was lower than 10 percent, which is weaker than its prediction gained from the second step till the fifth step. At the last step, LSTM_MidS could not predict the MidS correctly, where the highest probability in prediction is close to the FC, not to the MidS. LSTM_FC predicted the last FC correctly, however the highest probability of its prediction is lower than 10 percent.

Figure 7 outlines a short part of gait data from Test_1_G while the subject was walking, turning to the left, and walking back. As highlighted in the figure, the models correctly predicted all gait phases while the person was walking straight forward, however the models had some incorrect predictions while the subject was turning. Further, LSTM_FO predicted one FO at the first step of turning, LSTM_MidS estimated one MidS at the second step of turning and LSTM_FC detected one FC at the last step of turning. Since the subject was turning, the models must have ignored these gait phases. There were also some other incorrect predictions, however the level of probability was very low.

Figure 8 depicts a short part of the gait data from Test_3_cLBP while the subject was walking to the end of the track, turning, and then walking back. As Figure 8 shows, in most cases the models correctly predicted the gait phases during straight walking as well as during turning, except at the last step before turning and at the first step after turning. LSTM_FC did not detect the last FC before turning as well as the first one after turning. LSTM_FO also did not estimate the FO after the subject turned. LSTM_MidS had a better performance; however, it did not perfectly detect the first MidS after turning finished.

## 4. Discussion

In this study, three LSTM-based regression models were designed, trained, and validated in order to prove the possibility of using supervised deep learning algorithms, in particular LSTM algorithms for IMU-based gait analysis.

In general, manual labeling of gait data may be considered to be the most reliable approach for predicting the exact time point of gait phases. However, this approach may be highly time consuming and may not be an efficient way to analyze long-term gait data. Moreover, mathematical equations for gait phase estimation and gait segmentation can cause some meaningful errors when the data is noisy or when the person rarely reaches the steady state or changes the walking speed or walking style during the gait. Furthermore, recent advancements in artificial intelligence have made computers capable of learning and understanding patterns and similarities, which allows us to explore more about complex data. Therefore, it may be promising to involve this technology in gait analysis and overcome the weaknesses of currently available algorithms and reduce numerous lines of complex codes. The introduced approach in generating train dataset was a very simple but a very effective one. As mentioned before, the models were trained on gait data of only two persons and then validated and tested on data from other individuals having similar or different walking styles while performing similar or different tasks.

According to the results shown in Table 2 and Table 3, the accuracy and loss on train and validation datasets were clearly better than all test datasets. Since validation and test datasets were separated and contained different gait data, it may be possible to say that the models predicted more accurately when the data was similar to the train dataset. The acceptable performance of these models on test datasets proves the scalability of these LSTM-based models for gait phase detection. It also proves the capability of LSTM in learning the signal behavior of 251 frames (equal to 2.51 s) of gait data from three input features in order to predict the expected gait phases.

As mentioned before, the aim of designing these models was to estimate the closest time point for aforementioned gait phases considering all steps, including the first and the last step as well. Owing to the fact that the signals were first smoothed and then employed in the study, the time point of labeled gait phases were not exactly represented by the time point of gait phases proposed by [15]. However, as seen in Figure 2, the smoothed AngVelMag was a proper representative of the raw angular velocity employed in [15]. As seen in Figure 6, LSTM_FO could predict FO from the first two steps of the gait, despite the fact that the amplitude of FOs at these steps were lower than FCs, having quite a different signal shape compared to other steps during steady state of walking. Additionally, according to Figure 7 and Figure 8, the models could perfectly understand the turning stage of walking and avoid estimating gait phases.

Since these models return a time series of data comprising the probability (scaled from 0 to 1) of appearance of each gait phase for each time point of the signal, it is recommended to employ one of these models to first predict the most probable region that each gait phase could appear and then locally search for the exact time point of the occurrence of the desired gait phase from the raw AngVelMag or raw Gyr_Z based on [15], simply by employing peak detection algorithms and further straightforward lines of codes. As a result, each gait phase can be locally detected, which can reduce the incorrect estimations and increase the validity and reliability of automated IMU-based gait analysis.

According to our results, all three models performed well in predicting the desired gait phase, regardless of being trained on gait data collected from only two individuals. After proving our concept, it may be necessary to retrain such models by using more samples of data as well as improving the complexity of the neural network and optimizing hyperparameters to improve their performances for better results. This is very much the key component in future attempts to strengthen the estimation power of the proposed models. Available gait data may have limited us to testing the performance of our models on gait data of people suffering from other physical and neurological impairments. Future work may assess the capability of such models for more complex and abnormal gait patterns gained from multiple sclerosis, Parkinson’s disease patients and even cerebral palsy patients.

## Figures and Tables

**Table 1:** An overview of the datasets and some characteristics of the subjects.

Dataset	No. of Subjects	No. of Trials	Age (Mean ± std.)	BMI ^1^ (Mean ± std.)	No. of Sample Frames	No. of Labeled Frames for Each Gait Phase ^2^
Healthy individuals						
Train dataset	2	32	29.5 ± 4.9	23.8 ± 1.6	197,556	379
Healthy individuals						
Validation dataset	2	24	22.5 ± 0.7	21.5 ± 1	76,262	174
Healthy individuals						
Test_1_H	5	10	71 ± 7.2	27.6 ± 3.9	27,438	172
Test_2_H	11	22	71.4 ± 4.9	25.2 ± 2.4	118,422	514
Glaucoma patients						
Test_1_G	4	8	69.8 ± 4.1	26.7 ± 2.6	21,058	133
Test_2_G	7	14	71.1 ± 5.3	25.4 ± 3.6	86,050	341
cLBP patients						
Test_3_cLBP	37	74	68.9 ± 8.4	28 ± 4.6	260,054	1690

^1^ Body mass index; ^2^ each dataset had equal number of labeled FO, MidS and FC. The number of trials and samples frames and labeled frames is calculated by considering all data from both sensors.

**Table 2:** Accuracy and loss on train and validation datasets.

**Datasets**	**LSTM_FO** **Accuracy**	**LSTM_FO** **Loss**	**LSTM_MidS** **Accuracy**	**LSTM_MidS** **Loss**	**LSTM_FC** **Accuracy**	**LSTM_FC** **Loss**
Train	0.9981	0.0071	0.9981	0.0080	0.9981	0.0072
Validation	0.9977	0.0098	0.9977	0.0102	0.9977	0.0098

**Table 3:** Accuracy and loss on test datasets.

**Datasets**	**LSTM_FO** **Accuracy**	**LSTM_FO** **Loss**	**LSTM_MidS** **Accuracy**	**LSTM_MidS** **Loss**	**LSTM_FC** **Accuracy**	**LSTM_FC** **Loss**
Test_1_H	0.9937	0.0241	0.9937	0.0280	0.9937	0.0296
Test_1_G	0.9936	0.0322	0.9936	0.0303	0.9936	0.0395
Test_2_H	0.9957	0.0213	0.9957	0.0223	0.9957	0.0216
Test_2_G	0.9960	0.0186	0.9960	0.0192	0.9960	0.0199
Test_3_cLBP	0.9935	0.0284	0.9935	0.0309	0.9935	0.0343
Average	0.9945	0.0249	0.9945	0.0261	0.9945	0.0290
